# Expressed Sequence Tags as a Tool for Phylogenetic Analysis of Placental Mammal Evolution

**DOI:** 10.1371/journal.pone.0000775

**Published:** 2007-08-22

**Authors:** Morgan Kullberg, Björn Hallström, Ulfur Arnason, Axel Janke

**Affiliations:** Department of Cell and Organism Biology, Division of Evolutionary Molecular Systematics, University of Lund, Lund, Sweden; University of Dayton, United States of America

## Abstract

**Background:**

We investigate the usefulness of expressed sequence tags, ESTs, for establishing divergences within the tree of placental mammals. This is done on the example of the established relationships among primates (human), lagomorphs (rabbit), rodents (rat and mouse), artiodactyls (cow), carnivorans (dog) and proboscideans (elephant).

**Methodology/Principal Findings:**

We have produced 2000 ESTs (1.2 mega bases) from a marsupial mouse and characterized the data for their use in phylogenetic analysis. The sequences were used to identify putative orthologous sequences from whole genome projects. Although most ESTs stem from single sequence reads, the frequency of potential sequencing errors was found to be lower than allelic variation. Most of the sequences represented slowly evolving housekeeping-type genes, with an average amino acid distance of 6.6% between human and mouse. Positive Darwinian selection was identified at only a few single sites. Phylogenetic analyses of the EST data yielded trees that were consistent with those established from whole genome projects.

**Conclusions:**

The general quality of EST sequences and the general absence of positive selection in these sequences make ESTs an attractive tool for phylogenetic analysis. The EST approach allows, at reasonable costs, a fast extension of data sampling from species outside the genome projects.

## Introduction

In 1992 Novacek [1] presented a widely known hypothesis for the phylogenetic tree of placental mammals based on a synthesis of morphological and molecular findings. At that time only limited amounts of sequence data were available, a circumstance that rendered many ordinal relationships unresolved. During an initial stage phylogenetic analyses of sequence data were generally based on single genes or parts of genes [2]–[4]. This changed gradually and during the 1990's sequences of complete mitochondrial (mt) genomes became a common tool in phylogenetic analyses (e.g. [5], [6]). The combined sequences of all mt protein-coding genes yield alignment lengths of about 10–12 kbp, i.e. about 10-times the sequence amounts commonly used in the 1980s. However, in the absence of a closely related outgroup these analyses could not conclusively establish the direction of evolution in the placental tree. This limitation was amended by the first marsupial mt genome sequence, that of the opossum, *Didelphis virginiana* [7]. The marsupial rooting of the placental tree placed Rodentia (mouse, rat) as the sister group to remaining orders. This position of Rodentia was upheld in the great majority of the following mammalian mitogenomic (mtg) analyses, i.e. phylogenetic analyses based on the protein-coding genes of complete mt genomes (e.g. [8]–[12]). However recent mtg studies joined rodents and primates on a common branch (e.g. [13]). Thus, relationships within some basal parts of the placental tree remained equivocal, even in phylogenetic analyses of complete mt genomes. As some of these analyses demonstrated [9], [12], the basal position of the rodents in the mtg tree of placental mammals was sensitive to the sampling of other basal taxa and to the analytical approaches applied.

In 2001 Murphy et al. [14] presented phylogenetic results that challenged some parts of the placental mtg tree. The study was based on both mt data and directly PCR amplified introns of nuclear genes. The contribution of individual taxa to the complete data set differed somewhat and the alignment of the nuclear sequences showed considerable numbers of gaps and ambiguous sites. This was particularly noticeable in three of the nuclear sequences (≈ 50% of the nuclear data) in which the amino acid distance between human and the mouse ranged between 20% and 40%, a circumstance that may adversely affect the aligning of homologous sites. Similarly the concatenation of genes showing great evolutionary rate variation may affect the estimation of model parameters such as the gamma distribution parameter, α [15], [16]. The main parts of this nuclear gene tree [14] have nevertheless been supported in later studies based on far more comprehensive alignments [e.g. 17] and genome level characters like retroposon insertion and indel differences [18]–[20]. One of the main differences between this nuclear gene tree and previous mtg findings was that monophyletic Rodentia grouped with Lagomorpha, thereby supporting the morphological Glires hypothesis. Together with Primates, Dermoptera and Scandentia, Glires formed the super-ordinal clade Euarchontoglires. The sister group to the Euarchontoglires, called Laurasiatheria, included Artiodactyla, Carnivora and Perissodactyla among other orders. Euarchontaglires and Laurasiatheria are commonly joined in the Boreoeutheria.

The problems related to resolving basal placental relationships were again underlined in a recent study based on the sequences of eight housekeeping genes that were established by cDNA approaches from 22 placental mammals and three marsupials [21]. The total length of the alignment was 6 kb and all genes had similar evolutionary rates. Inconsistent with the results of Murphy et al. [14] the analyses favored a tree with Glires in a basal position relative to Primates rather than joining Primates and Glires on a common branch. Furthermore, and despite an extended and more uniform sequence representation of each individual taxon as compared to the study of Murphy et al. [14], the position of the root of the placental tree was not conclusively established when rate heterogeneity models were applied [21].

In this study we have selected the Boreoeutheria group to examine the utility ESTs for phylogenetic analyses, as a novel approach to economically obtain large amounts of protein-coding sequences. The procedure rests upon the random retrieval of ESTs from a cDNA library, which represents all expressed genes in a cell at a given time [22]. The ensuing database search allows subsequent complementation with orthologous sequences from species that are of interest to the phylogenetic study. This approach has hitherto been applied in only a limited number of phylogenetic studies that have addressed deep relationships among eukaryots, plants, arthropods and mammalians [23]–[27].

Currently, genome projects of some 20 placental and marsupial mammals are in different stages of completion. Sequence data from these projects have allowed resolution of several ordinal and superordinal placental relationships [17] with which the results from the EST based phylogenetic analysis can be compared. In order to promote the identification of the root of the placental tree we have, along with the production of placental sequences established the homologous sequences from an Australian marsupial, the fat-tailed dunnart, *Sminthopsis crassicaudata*. With an upper paleontological limit of about 125 million years before present, MYBP, for the divergence between marsupial and placental mammals [28] the inclusion of *Sminthopsis* constitutes a definite advantage in determining the root of the tree of placental mammals.

## Results

More than 1.200.000 nt sequences representing about 2000 EST sequences were retrieved from the *Sminthopsis* tissue culture cells (fibroblasts). About 1600 EST sequences with a minimum length of ≈400 bp were collected for further evaluation. After excluding vector and mt sequences, 854 individual nuclear cDNA sequences and contigs remained for the complementary database search. Orthology search against the human mRNA RefSeq database identified 455 protein-coding sequences with E-values <10^−15^ that were subsequently aligned. A list of the accession numbers of the putative 455 human orthologous mRNA sequences is provided in the Table S1. Several un-translated sequences were identified during the search. These sequences were not included in the study as it focuses on protein-coding genes. 344 of the 455 human mRNA transcripts could be classified according to the PANTHER classification system, while 109 sequences remained unclassified. Table 1 shows the classification for those gene classes that had more than five members.

**Table 1:** Classification of the human homologues

Function	Number of genes
Protein biosynthesis	71
Transport related	35
mRNA Transcription	27
Cell structure	22
Proteolysis	21
Cell cycle	17
Protein folding	16
mRNA splicing	13
Protein phosphorylation	12
Nucleoside, nt and na metabolism	10
DNA replication	10
Stress response	8
Cell motility	8
rRNA metabolism	8
Mitosis	8
Protein modification	7
Oxidative phosphorylation	7
Tricarboxylic acid pathway	6
Cell adhesion	6
Immunity and defense	6
Protein complex assembly	6
Protein glycosylation	6
Cell communication	6
Developmental processes	6
Intracellular protein traffic	6
Unclassified	109

NOTE–Only the most common classes according to the PANTHER classification with >5 identified homologues are shown.

Of these 455 sequences a total of 161 sequences were represented by seven placental species (elephant, mouse, rat, rabbit, human, cow and dog). This alignment (named *maxspe*) that maximized the mammalian representation for all sequences had a length of 77,328 nt (25,776 aa). A second alignment that maximized the number of sequences by allowing some sequences to be missing was also constructed. This alignment, which included 326 sequences (164,466 nt or 54,822 aa) from the eight species, is referred to as the *maxgen* alignment. Genomes with a low current sequencing coverage such as those of the elephant and the rabbit were allowed to lack 25% of the genes. In a few cases one or two sequences of cetferungulates (cow or dog) and/or rodents (mouse or rat) were allowed to be missing in the *maxgen* alignment. The chicken was not represented in about 33% of the alignments for both *maxspe* and *maxgen* and was therefore excluded from all analysis based on single genes. The general properties of the two datasets are given in Table 2.

**Table 2:** General statistics of the concatenated data sets

Data set	Length nt	Gaps	Distance human-mouse			Constant sites		
			123cdp	12cdp	aa	123cdp	12cdp	aa
maxgen	164466	54%	0.111	0.033	0.050	27.2%	34.2%	32.1%
maxspe	77328	6%	0.113	0.036	0.055	55.1%	69.4%	65.1%

NOTE–The observed percentages and distances (substitutions per site) are shown.

**Figure 1:**
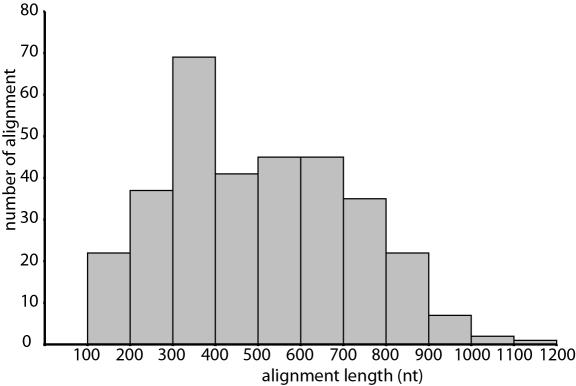
Distribution of the alignment lengths after trimming.

**Figure 2:**
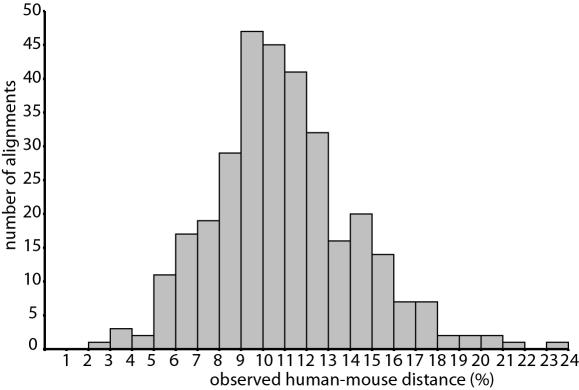
Distribution of the pair wise aa distances between human and mouse.

The length of the individual and trimmed alignments excluding gaps varied from 126 to 1167 nt, with an average of 505 nt (Figure 1). The genetic distances between human and mouse ranged from 0% to 20% (mean = 4.5±4.5) for aa sequences (Figure 2), 0%–18% (mean = 3.2±2.9) for first and second codon positions (12cdp), and 3%–24% (mean = 11.0±3.3) for all codon positions (123cdp). Alignments with zero aa distance between human and mouse or human and cow were excluded from the analysis. Additional properties such as the number of gaps and number of constant sites in the different alignments are shown in Table 2.

Estimates of potential sequencing errors in *Sminthopsis* ESTs indicated an error rate of approximately 0.01% and allelic variation of about 0.02%. Further evidence that sequence differences had been correctly classified as allelic variation rested on the observation that the sequence differences occurred always at silent 3rd codon positions. Most of the differences constituted frequent naturally occurring C-T transitions. A potential error rate of 0.01% was also recorded in 102,232 nt of mt ESTs with a 10-fold coverage of about 10,000 nt of overlapping mt protein-coding sites. Comparison between the EST data and the mt genome of another *Sminthopsis* individual showed 134 differences (0.1%). This value is within the expected sequence variation of mt sequences of different individuals. The results suggest that sequence differences related to sequencing errors are less frequent than natural allelic variation, although the statistics behind these differences is limited due to the low total numbers of differences. The findings suggest that potential sequencing errors in the EST sequencing study are at a level that effects the current phylogenetic analyses far less than allelic variation.

The aa distances within the two alignments, *maxgen* and *maxspe*, are shown in Table 3. Distances between the outgroup and the ingroup taxa differed by ≈ 10%, indicating a limited difference in evolutionary rates among the ingroup species. There is a notable difference between the marsupial and placental distances relative to the chicken, indicating a faster evolution in the placentals. Among the placental mammals the sequences of Glires and the elephant appear to evolve faster than those of *Homo* and the cetferungulates. A chi-2 test as implemented in TREE-PUZZLE showed that composition of the aa as well as 1st and 2nd codon positions (12cdp) is homogeneous among the mammalian species. However, nt composition was not homogeneous when the same test was applied to all three codon positions.

**Table 3:** Pairwise aa distances between species.

	Chicken	*Sminthopsis*	Elephant	Dog	Cow	Human	Rabbit	Mouse	Rat
Chicken		0.108	0.126	0.113	0.116	0.111	0.125	0.117	0.118
		0.135	0.162	0.142	0.146	0.139	0.161	0.147	0.149
*Sminthopsis*	0.125		0.103	0.092	0.093	0.090	0.103	0.100	0.100
	0.160		0.126	0.110	0.112	0.108	0.126	0.122	0.123
Elephant	0.141	0.108		0.051	0.056	0.050	0.064	0.067	0.068
	0.184	0.133		0.056	0.062	0.054	0.073	0.075	0.077
Dog	0.125	0.096	0.054		0.037	0.036	0.047	0.053	0.054
	0.158	0.114	0.059		0.039	0.038	0.051	0.058	0.059
Cow	0.128	0.100	0.059	0.039		0.040	0.052	0.057	0.058
	0.162	0.120	0.65	0.042		0.042	0.057	0.063	0.064
Human	0.124	0.096	0.053	0.038	0.043		0.046	0.050	0.050
	0.157	0.113	0.058	0.040	0.046		0.050	0.064	0.055
Rabbit	0.135	0.108	0.066	0.050	0.056	0.048		0.060	0.062
	0.176	0.132	0.074	0.054	0.061	0.052		0.066	0.070
Mouse	0.132	0.106	0.072	0.058	0.063	0.055	0.048		0.020
	0.170	0.129	0.082	0.064	0.069	0.060	0.071		0.021
Rat	0.133	0.108	0.074	0.060	0.064	0.056	0.066	0.023	
	0.171	0.132	0.084	0.066	0.071	0.062	0.071	0.024	

Note—The values show the observed and JTT+4Γ+Ι distances (top to bottom). Above diagonal the *maxgen* and below *maxspe* alignment.

**Figure 3:**
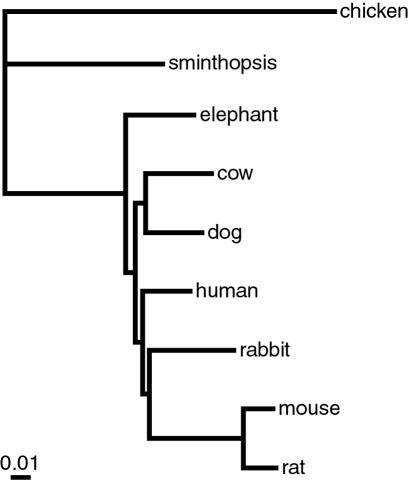
Phylogenetic relationships based on partitioned Bayesian inference of the *maxgen* alignment (164466 nt) and a GTR+4Γ+I model of sequence evolution. The partitioning are according to four evolutionary rate classes and codon positions. The model parameters are estimated separate for every partitioning.

Figure 3 shows the Bayesian tree based on the nt sequences of the *maxgen* dataset. Posterior probability values were 1.00 for all nodes in the tree. ML rate heterogeneity bootstrap support from the *maxspe* data was moderate to high (70–100%) for the aa and cdp123 but low for cdp12 (39% for Boreoeutheria). When the chicken, the elephant, and the rabbit were excluded from the alignment there was a 0.85 posterior probability for the Euarchontoglires clade. Unpartitioned ML analyses including all species resulted in the same topology as in Figure 3, but rodents fell basal when the chicken, the elephant, and the rabbit were excluded. NJ and MP analysis generally placed rodents as sister group to all other placentals, regardless of the taxon sampling.

Likelihood ratio tests showed that partitioning of the data significantly improved the fit to the evolutionary model. The largest impact was seen from partitioning according to codon positions, where the increase in the logL values was several thousand. Partitioning according the genetic distances increased the logL values by a few hundred logL values, which was still a statistically significant improvement.

**Figure 4:**
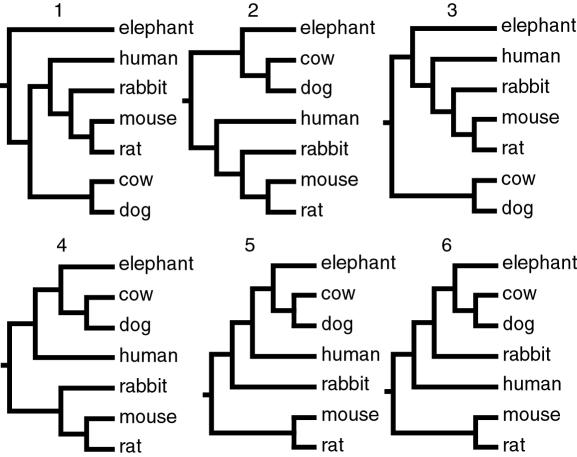
Relationships between species in the tested alternative topologies.

In order to further investigate the support for the best tree shown in Figure 3 the ΔlogL/S.E. ratio and the pSH values were calculated for alternative placental trees, Figure 4. The alternative trees represented the 2nd and 3rd best ML alternatives according to PROTML, an alternative tree based on housekeeping genes [21], the MP and NJ aa tree and the mtg tree [9]. Different evolutionary models were used to calculate the likelihood and pSH values for the *maxgen* and *maxspe* alignments on the basis of aa, 12cdp and 123cdp nt sequences (Table 4). All analyses identified tree-1 as the best tree, but alternative topologies could not be statistically rejected by all datasets, except in the case of tree-4.

**Table 4:** ML support for the different phylogenetic hypothesis.

			tree-1	tree-2	tree-3	tree-4	tree-5	tree-6
aa	*maxgen*	JTT+4Γ+I	[250468.7]	2.11	1.63	3.03	2.20	2.21
		pSH	1.000	0.313	0.417	0.010	0.020	0.019
	*maxspe*	JTT+4Γ+I	[127791.9]	0.96	0.67	2.15	1.81	1.68
		pSH	1.000	0.670	0.673	0.090	0.052	0.060
12cdp	*maxgen*	GTR+4Γ+I	[331163.9]	2.59	2.53	3.77	1.86	1.63
		pSH	1.000	0.216	0.248	0.000	0.033	0.067
	*maxspe*	GTR+4Γ+I	[152577.9]	1.43	1.79	2.23	1.08	0.89
		pSH	1.000	0.482	0.404	0.072	0.203	0.259
123cdp	*maxgen*	GTR+4Γ+I	[668754.0]	6.84	6.03	5.63	1.18	2.65
		pSH	1.000	0.001	0.006	0.000	0.151	0.007
	*maxspe*	GTR+4Γ+I	[326172.5]	4.33	5.35	4.47	1.93	3.39
		pSH	1.000	0.056	0.069	0.000	0.035	0.000

Note–The support is expressed as the ΔlogL/S.E. ratio and the pSH value. The -logL value of the best tree is shown in square brackets.

The 161 genes of the *maxspe* alignment were individually analyzed in order to estimate the proportion of genes that supported each of the six alternative topologies shown in Figure 4. The logL for each of these topologies was calculated using TREE-PUZZLE. If the ΔlogL/S.E. ratio value between alternative trees exceeded 0.5 the topology was recorded. If the value was less than 0.5, the support was regarded as inconclusive. Although the cut-off ΔlogL/S.E. ratio of 0.5 was arbitrarily chosen, ΔlogL/S.E. ratios of this level indicate some support for the best topology over alternatives. Table 5 summarizes the support for alternative trees as recorded for individual genes applying the different analytical approaches. Most genes that contained enough phylogenetic information to distinguish between alternative topologies supported tree-1. The strongest phylogenetic support came from single gene analyses of the 123cdp alignments. However, the majority of the single genes does not carry sufficient phylogenetic information to distinguish between the alternative trees.

**Table 5:** Support of three alternative topologies by ML analysis of single genes.

Topology	Number of genes		
	aa+4Γ+I	cdp12+4Γ+I	cdp123+4Γ+I
tree-1	25	31	42
tree-2	3	0	4
tree-3	14	16	23
tree-4	4	8	2
tree-5	12	7	11
tree-6	11	20	17
unresolved	92	79	62

NOTE–The number of genes that support a fixed topology with a ΔlogL/S.E. ratio equal or better than 0.5 are shown. The resolution was regarded as unresolved when the ΔlogL/S.E. ratio was smaller than 0.5.

The nt and aa sequences of single genes from the *maxspe* dataset were analyzed with respect to their support for internal branches by calculating and comparing the ML values of different trees using PHYML and quartet puzzling (QP) as implemented in TREE-PUZZLE. The CONSENSE program (PHYLIP) was used to summarize the gene-trees by calculating a majority rule consensus tree. Table 6 shows the number of genes that favored selected internal branches according to the ML and QP analyses. Depending on the mode of analysis the support for different clade varied. The only branch receiving strong support was Rodentia, represented by the relatively closely related mouse and rat; all other branches were weakly supported, notably by the QP method.

**Table 6:** Numbers of genes that support a certain clade.

Clade	Number of genes		
	aa+4Γ+I	12cdp+4Γ+I	123cdp+4Γ+I
Boreotheria	13/55	14/49	24/50
Euarch.glir	7/49	7/31	14/58
Glires	17/61	18/50	21/54
Rodentia	111/147	128/142	158/160
Cetferung.	23/74	22/60	39/82

NOTE–The trees have been reconstructed for single gene alignments with TREE-PUZZLE (first value) and PHYML (second value) respectively. HDC: human plus dog plus cow clade, Euarch.glir: Euarchontaglires clade (human plus rodents).

The effect of taxon sampling and the choice of outgroup species on the phylogenetic reconstruction were evaluated by analyzing the data after excluding one or more species at the time. Inclusion of the chicken as an extra outgroup besides *Sminthopsis* has no effect on the analysis, but when only the chicken was used as outgroup tree-1 and tree-5 became nearly indistinguishable. Likewise exclusion of rabbit led to the promotion of a basal position of rodents as in trees 4, 5 and 6. Euarchontaglires remained supported after exclusion of the elephant (trees 1, 2 and 3). Exclusion of either mouse or rat, or cow or dog had no effect on the topology.

In order to investigate whether directional (positive) selection might have affected the tree reconstruction, a ML analysis applying a branch-site model of evolution as implemented in PAML was performed. The analysis was carried out both on the individual genes of the *maxspe* dataset and the concatenated *maxgen* data. Among the single genes, 22 had at least one branch with codons that had ω >1, Table 7. This suggests that only a few genes have single sites that might be affected by positive selection. Analyses of the concatenated *maxgen* dataset confirmed that most branches have only a few single codons with ω >1 that are under selection, Table 7. The concatenated sequences of the elephant appeared to contain more sites (40) under positive selection than in the other mammalian species studied.

**Table 7:** Sequences showing signs of positive selection on at least one branch under branch-site models. Significant LRT at 1%.

Human accession number	Branch/Species	Codon positions under pos. selection/number of codons	Gene function/name
NM_001064	elephant	2/204	transketolase
NM_003670	human	1/132	basic helix-loop-helix
NM_005094	cow	1/86	solute carrier
NM_005866	rabbit	1/126	opioid receptor
NM_015150	Sminthopsis	2/76	lipid raft linker
NM_012225	rabbit	2/163	nucleotide binding protein
NM_000546	rabbit	3/123	tumor protein p53
NM_004559	elephant	5/243	Y box binding protein
NM_001428	rat	1/284	p53-binding protein
NM_006739	rabbit	1/216	minichromosome maintenance complex
NM_018622	rat	11/207	presenilin associated
NM_000989	cow	4/114	ribosomal protein L30
NM_024092	elephant	2/182	transmembrane protein
NM_006449	dog	2/200	CDC42 effector protein
NM_018285	dog	2/172	ribonucleoprotein
NM_005530	cow	2/232	isocitrate dehydrogenase
NM_015292	cow	1/265	family with sequence similarity 62
NM_004135	cow	1/199	isocitrate dehydrogenase
NM_018361	rodentia	2/108	lysophosphatidic acid acyltransferase
NM_006530	rabbit	2/218	YEATS domain containing 4
NM_002306	rabbit	2/182	galactoside-binding
NM_002306	mouse	1/182	galactoside-binding
NM_014300	rat	1/163	SEC11 homolog A
Concatenated	Chicken	55/28521	
Concatenated	*Sminthopsis*	8/28521	
Concatenated	Elephant	40/28521	
Concatenated	Cow	7/28521	
Concatenated	Dog	3/28521	
Concatenated	Human	1/28521	
Concatenated	Rabbit	5/28521	
Concatenated	Mouse	1/28521	
Concatenated	Rat	4/28521	
Concatenated	Cetferungulata	1/28521	
Concatenated	Rodentia	1/28521	
Concatenated	Glires	0/28521	
Concatenated	Euarchontaglires	0/28521	
Concatenated	Boreotheria	0/28521	
Concatenated	Placentalia	9/28521	

## Discussion

Phylogenetic studies of deeper mammalian relationships, such as those of placental orders, are to a great extent based on sequence analysis of protein-coding genes. Compared to the protein-coding regions the non-coding regions of the genomes evolve in general much faster and are often rearranged and randomized by multiple substitutions. Hitherto, nuclear gene sequences used in phylogenetic analyses have commonly come from genomic PCR-amplifications of single exons or from intron-less protein-coding genes (e.g. [14], [29], [30]). The application of cDNA approaches circumvents this limitation as it allows the production of complete coding sequences from a variety of genes [21]. Sequencing of ESTs is one method to economically produce large amounts of protein-coding sequences for phylogenetic purposes.

About 45% of the EST sequences obtained in the current study constituted multiple nuclear sequences and about 7% were mt encoded. About 1/3rd of the nuclear sequences and contigs had putative homologues among the selected species and could be used for phylogenetic analysis. Most of the ESTs produced in the current study represented genes that can be classified as housekeeping genes [31]–[33]. Among mammalian orders the aa distances of housekeeping genes are generally limited to only a few percent, making them an ideal choice for phylogenetic analysis due to the facility with which correct alignments can be established. The limited distances among homologous housekeeping genes contrasts in this respect to nuclear genes such as vWF (von Willebrand factor), IRPB (interphotoreceptor retinoid binding protein) and the BRCA1 (breast and ovarian cancer susceptibility protein 1) sequence, which are commonly included in phylogenetic analyses. These three sequences have aa distance of 20–45% among mammalian orders. In comparison to these three sequences the effect of randomization in housekeeping genes can be considered as being limited. While it can be argued that the conserved nature of the housekeeping genes reduces the phylogenetic content of each single gene, this is compensated by the large amount of different EST sequences that can be produced from each individual taxon. Another advantage of applying cDNA sequences is that the risk of including pseudogenes is low. Although there are reports suggesting that some pseudogenes are transcribed [34], it is conceivable that pseudogenic sequences, if present, are rare among the large number of genic EST sequences. Thus, even if a few pseudogenes might occur among the ESTs they would be expected to have little or no influence on the phylogenetic outcome. By applying search against mRNA databases such as RefSeq the potential inclusion of pseudogenic sequences is also counteracted.

One particular difficulty in any phylogenetic study that utilizes nuclear encoded genes is the establishment of their orthology. When PCR based approaches are used to amplify sequences from genomic DNA [14], [29], [30] or cDNA [21] the orthology can only be assumed by similarity criteria. Criteria that use phylogenetic information [25] are generally preferred, but these depend on a known tree, the very subject that is under study. Synteny is another powerful criterion that has been used to define orthology [34]. Determination of orthology by the way of synteny analysis could not be achieved in the current study since it requires that the sequence of almost the whole genome is available, which is not the case for *Sminthopsis*. For this reason a number of precautions were undertaken in the current study in order to minimize the risk of including paralogous or pseudogenic sequences. This included reciprocal BLAST searches between human and the other species, with a cutoff E-value of 10^−15^ in order to ensure orthology between species. Also the use of a high quality databases such as RefSeq promotes the inclusion of known functional genes rather than pseudogenes in the analysis. In addition, after finding a possible human homologue, its full-length cDNA sequence instead of the shorter EST sequences was used for reciprocal searches in other species to further increase the chance of identifying orthologous genes in other species. The rigorous approach applied aims to maximize the probability of including only orthologous genes in the analyses.

There have been concerns about the quality of EST data for phylogenetic analyses [35], because most of the individual sequences are based on single reads. This study showed, however, that sequence differences due to sequencing errors of ESTs are at a level similar to that of allelic variation or even lower. The potential effect of errors of this kind can therefore be considered as negligible for the current phylogenetic results.

The phylogenetic analyses appeared stable with respect to the assumption of evolutionary neutrality. The search for positive selection identified only few genes with single sites that had an ω value >1 in one or more branches. Other studies [36] have identified even fewer incidents of positive selection, when a pairwise method [37] was applied to compare ω between distant groups from different animal classes. The pairwise approach of that study probably identified fewer candidate genes, because the evaluation did not rely on phylogenetic information. Thus, selection may have been active on single branches in the past without the signal being recognized. The discrepancy between the number of sites identified by the analysis of the single genes and the concatenated *maxgen* dataset may be related to more robust statistics when larger numbers of codons are involved in the analysis.

Compared to the total number of characters the low number of codons and the few genes that appear to be under selection appear to have no practical effect on the phylogenetic reconstruction. Only a few branches are affected by positive selection and the selection was not specific for a particular group, species or branch. If the phylogeny is incorrect, however, e.g. rodents or Glires as sister to the remaining species as in tree-4 to tree-6, fewer sites should actually be selected for, because in suboptimal phylogenies the number of sites under selection becomes over-estimated [38].

Phylogenetic examination of the eight placental taxa that are represented by nearly complete genomes joined primates and Glires as sister groups composing the clade Euarchontoglires. This clade is sister to Boreoeutheria as represented by the cow and the dog. The analyses favored a sister group relationship between the elephant and all other placentals included. The same general topology was also found using sequences from the ENCODE consortium [18], [39]. Other studies [14], [19], [40] have favored the same topology using shorter alignments but a more comprehensive taxon sampling. These results challenge previous studies using EST [24], large-scale genomic data [41]–[43], mtgs [9] and the analysis of few housekeeping genes [21].

Basal mammalian divergences have proven difficult to resolve, despite the use of large amount of nuclear sequences. This may reflect the potentially narrow temporal window within which the divergences took place, leaving only a low number of phylogenetically informative sites. While this constitutes a limitation that is common to all phylogenetic analyses the impact that taxon sampling may have on the tree is striking. Thus, in the current study Rodents tended to become the sister group to other placentals when the rabbit was not included. The same tendency, which may be the effect of long branch attraction [44], has been observed earlier is studies with a limited taxon sampling [41]–[43]. An indication of a long branch attraction between rodents and the outgroup is that tree-5 was favored by MP and NJ analyses. Especially MP is known to be sensitive to evolutionary rate differences among the taxa and long branch attraction [44].

Among placental mammals the rodents may behave as a so-called rouge taxon, i.e. a lineages that tends to skew phylogenetic analyses [45]. This effect can be overcome by excluding taxa of this kind [46] or by including less deviating taxa for compensation. In the current study the attraction between rodents and the outgroup could be compensated by the inclusion of lagomorph sequences and complex model of sequence evolution. Other studies have questioned the use of long branch attraction phenomenon as an explanation to a basal position of rodents, because this particular topology received strong support from slowly evolving genes, while fast evolving genes supported Euarchontaglires [43]. The authors concluded that this contradicts the expectation from the effect of long branch attraction. The tendency of ML analysis to join fast (mouse) and slow (human) evolving taxa has instead been coined “long branch repulsion” or “opposite branch attraction” [43]. It is not clear how effects of this kind may have affected the current results, because it is not obvious why the rabbit would promote the opposite branch attraction phenomenon. The shift in the topology favored by ML when the rabbit is excluded form the analysis is more easily explained by long branch attraction between rodents and the outgroup. Further evidence for long branch attraction comes from the observation that ML cannot distinguish between tree-1 and tree-5 when only chicken is used as an outgroup. It appears that in these analyses the rodents are dragged towards the root of the placental tree. This illustrates the importance of choosing a not too distant outgroup and justifies the establishment of the marsupial EST sequences for this study.

Assumptions about evolutionary models have a major impact on the recovered phylogeny. A parameter rich model naturally fits the data better than a simpler model [47]. Dividing the data into partitions increases the number of parameters and thereby the fitness of the model. In order to create evolutionary models that are more realistic Kjer and Honeycutt [40] partitioned the sites in a mtg analysis of eutherian relationship into classes according to their relative MP consistency index. This resulted in a mtg phylogeny with strong support for the Atlantogenata hypothesis, which has not being supported by non-partitioned mtg analysis [9]. The performance of different partition strategies for concatenated data has been studied for mollusk sequence data. Partitioning data according to codon positions, and to a lesser extent also by genes, improves the fitness of models to the data [16]. In a large and variable dataset such as in this EST study, one can expect that extensive rate heterogeneity that may not be correctly accounted for by using non-partitioned analyses.

We tried to account for among site rate variation by partitioning the data into codon positions and also after the evolutionary rate of the genes. Obviously, partitioning according to single genes was impossible due to the number of genes included in this study. The size of each partition needs also to be reasonable large in order to get an accurate estimations of each parameter. We therefore divided the data according to distance classes and codon positions. Our approach to account for the among site rate variation satisfy the desire for a realistic model and still keeps the analysis on a computational acceptable level.

As mentioned above a limited taxon sampling may lead to an incorrect topology due to assuming models that are not consistent with the evolution. This could explain the results of some previous studies [24], [41], [42]. The strong attraction of rodent to the outgroup disappears for our data when they are partitioned and analyzed under Bayesian approaches.

While complete genomes are the ultimate data sets for resolving phylogenetic and evolutionary issues of different kinds (e.g. [28], [41]), the costs of producing these data sets are still at a level that that precludes a dense taxonomic sampling among higher organisms. There is therefore a need to establish methods that at reasonable costs allow the production of sequence data that can be of general interest for phylogenetic studies. Producing EST sequences is such a method that will gain more attention in the future.

## Materials and Methods

Total RNA was isolated from fibroblast cell culture (EAECC number: SC 11) of *Sminthopsis crassicaudata* (fat-tailed dunnart) using the acid phenol guanidinium thiocyanate method, GTC-method [48]. Enriched mRNA was reverse transcribed, size fractionated, and cloned, yielding a total of 2000 cDNAs that were sequenced by QIAGEN (Germany) (Accession number EV533153-EV534821). The retrieved sequences were analyzed for identical or overlapping sequences from different clones using the Sequencher version 4.6 (Gene Codes) software. Contigs were assembled from overlapping genes. The sequencing error rate and the proportion of allelic variation were estimated by comparing more than 500 nucleotides (nt) from ESTs that were represented by two or more clones. Nt differences were recorded as allelic variation when at least two different nt occurred at the same site, with each type being represented in at least two sequences. Other differences were counted as potential sequencing errors. As evident with this approach allelic variation becomes automatically underestimated and sequencing errors overestimated unless comprehensive sequence coverage exists. Furthermore, the sequence error rate was estimated from comparing mt EST sequences among themselves and to the complete mt genome from another individual (Accession number NC_007631).

Individual sequences and contigs were used to search for homologous sequences in the mRNA database using blastn as implemented in the EST-e-mate v1.0 program package (in-house application, code available from authors Hallström and Janke). In short, EST-e-mate blasts the marsupial ESTs against the NCBI human mRNA RefSeq database [49]. The human sequences with the lowest E-value (expect value) were chosen as a template for searching for homologous sequences from the corresponding RefSeq database [49] of chicken (*Gallus gallus*), mouse (*Mus musculus*), rat (*Rattus norvegicus*), cow (*Bos taurus*) and dog (*Canis familiaris*) as at 30.Apr.2006. Rabbit (*Oryctolagus cuniculus*) and elephant (*Loxodonta africana*) were retrieved from ENSEMBL [50] 1.Apr.2007. Species which represent very close relatives (e.g. chimpanzee to human) were not included in the analysis. The elephant was chosen, because it shows the slowest evolutionary rate among the non-boreoeutherians [17]. Sequence hits with E-values above 10^−15^ were excluded from further analysis. The program EST-e-mate utilizes ClustalW [51] for aligning the sequences, while keeping the reading frame intact with sequence alignments trimmed relative to the shortest sequence. Gaps and columns with ambiguous characters were removed. Potentially faulty alignments, i.e. alignments in which any taxon pair had amino acid (aa) distance value >0.6 were inspected further. All alignments were manually inspected using the Se-Al v2.0a11 software [52] and analyzed individually or as concatenated files. The mRNAs were functionally classified using the PANTHER classification system [53]–[54].

Sequence data were analyzed using the TREE-PUZZLE [55], PHYLIP [56], MOLPHY [57], MrBayes v3.1.2 [58], PAML3.15 [59], TREEFINDER [60], PHYML [61] or PAUP* [62] program packages. For the concatenated data the best-fitted model for nt sequence evolution and parameters were determined applying MODELTEST version 3.7 [63] and PROTTEST version 1.3 [64]. The JTT model [65] of amino acid (aa) sequence evolution and the GTR model [66] of nt evolution were used for distance and likelihood analyses. All phylogenetic analyses were computed assuming a gamma model of rate heterogeneity [67] with four classes of variable sites and one class of invariable site (4Γ+I). When a program did not allow for invariable sites, eight classes of variable site were used (8Γ). For the *maxgen* alignment Bayesian analysis were conducted running two simultaneously analysis with MrBayes applying one cold and three heated chains for 10,000,000 MCMC (Markov chain Monte Carlo) generations, discarding the first 1,000,000 generations as burnin. To compensate for the rate heterogeneity in the data we divided the alignment into twelve partitions, each with its own individual GTR matrix, gamma distribution, proportion of invariable sites and base frequencies. The four main partitions were according to the observed (0–5%, 5–10%, 10–15%, >15%) aa distances between human and mouse. These four partitions were further divided according to codon positions. For aa and cdp12 the dataset were divided only into four partitions according to aa distances. Bayesian analyses were made on the TITAN cluster of the Bioportal [68].

Analyses for potential selection were made for the concatenated sequences and single genes using codeml (PAML3.15) by estimating the non-synonymous (dN), synonymous (dS) substitution rates and ω (dN/dS) for one branch at a time. This approach corresponds to the branch-site model. In this model the branch of interest (foreground) can have sites with an ω-value larger than one and all other branches (background) are restricted to ω-values below or equal to one [69]. A Bayes empirical Bayes (BEB) procedure [70] was used to identify the sites evolving under potential positive selection. The codon frequencies were estimated from the data (CodonFreq = 3). All alignment columns containing ambiguities and gaps were excluded during the PAML analysis.

## Supporting Information

Table S1 Accession numbers of 455 the human genes homologous to the ESTs
(0.12 MB DOC)

## References

[1] Novacek MJ (1992). Mammalian phylogeny: shaking the tree.. Nature.

[2] de Jong WW, Zweers A, Goodman M (1981). Relationship of aardvark to elephants, hyraxes and sea cows from a-crystallin sequences.. Nature.

[3] Miyamoto MM, Goodman M (1986). Biomolecular systematics of eutherian mammals: phylogenetic patterns and classification.. Syst Zool.

[4] Irwin DM, Kocher TD, Wilson AC (1991). Evolution of the cytochrome b gene of mammals.. J Mol Evol.

[5] Arnason U, Johnsson E (1992). The complete mitochondrial DNA sequence of the harbor seal, Phoca vitulina.. J Mol Evol..

[6] Adachi J, Cao Y, Hasegawa M (1993). Tempo and mode of mitochondrial DNA evolution in vertebrates at the amino acid sequence level: rapid evolution in warm-blooded vertebrates.. J Mol Evol.

[7] Janke A, Feldmaier-Fuchs G, Thomas WK, von Haeseler A, Pääbo S (1994). The marsupial mitochondrial genome and the evolution of placental mammals.. Genetics.

[8] Arnason U, Gullberg A, Janke A, Xu Y (1996). Pattern and timing of evolutionary divergences among hominoids based on analyses of complete mtDNAs.. J Mol Evol.

[9] Arnason U, Adegoke JA, Bodin K, Born EW, Esa YB (2002). Mammalian mitogenomic relationships and the root of the eutherian tree.. Proc Natl Acad Sci U S A.

[10] Cao Y, Waddell PJ, Okada N, Hasegawa M (1998). The complete mitochondrial DNA sequence of the shark Mustelus manazo: evaluating rooting contradictions to living bony vertebrates.. Mol Biol Evol.

[11] Reyes A, Pesole G, Saccone C (2000). Long-branch attraction phenomenon and the impact of among-site rate variation on rodent phylogeny.. Gene.

[12] Arnason U, Janke A (2002). Mitogenomic analyses of eutherian relationships.. Cytogenet Genome Res.

[13] Reyes A, Gissi C, Catzeflis F, Nevo E, Pesole G (2004). Congruent mammalian trees from mitochondrial and nuclear genes using Bayesian methods.. Mol Biol Evol.

[14] Murphy WJ, Eizirik E, O'Brien SJ, Madsen O, Scally M (2001). Resolution of the early placental mammal radiation using Bayesian phylogenetics.. Science.

[15] DeBry RW (2003). Identifying conflicting signal in a multigene analysis reveals a highly resolved tree: the phylogeny of Rodentia (Mammalia).. Syst Biol.

[16] Strugnell J, Norman M, Jackson J, Drummond AJ, Cooper A (2005). Molecular phylogeny of coleoid cephalopods (Mollusca: Cephalopoda) using a multigene approach; the effect of data partitioning on resolving phylogenies in a Bayesian framework.. Mol Phylogenet Evol.

[17] Nikolaev S, Montoya-Burgos JI, Margulies EH, Program NCS, Rougemont J, Nyffeler B, Antonarakis S (2007). Early History of Mammals Is Elucidated with the ENCODE Multiple Species Sequencing Data.. PloS Genet.

[18] Kriegs JO, Churakov G, Kiefmann M, Jordan U, Brosius J, Schmitz J (2006). Retroposed elements as archives for the evolutionary history of placental mammals. PLoS Biol..

[19] Murphy WJ, Pringle TH, Crider TA, Springer MS, Miller W (2007). Using genomic data to unravel the root of the placental mammal phylogeny.. Genome Res.

[20] Waters PD, Dobigny G, Waddell PJ, Robinson TJ (2007). Evolutionary History of LINE-1 in the Major Clades of Placental Mammals.. PloS ONE.

[21] Kullberg M, Nilsson MA, Arnason U, Harley EH, Janke A (2006). Housekeeping genes for phylogenetic analysis of eutherian relationships.. Mol Biol Evol.

[22] Adams MD, Kelley JM, Gocayne JD, Dubnick M, Polymeropoulos MH (1991). Complementary DNA sequencing: expressed sequence tags and human genome project.. Science.

[23] Philippe H, Snell EA, Bapteste E, Lopez P, Holland PW (2004). Phylogenomics of eukaryotes: impact of missing data on large alignments.. Mol Biol Evol.

[24] Jorgensen FG, Hobolth A, Hornshoj H, Bendixen C, Fredholm M (2005). Comparative analysis of protein coding sequences from human, mouse and the domesticated pig.. BMC Biol.

[25] de la Torre JE, Egan MG, Katari M, Brenner ED, Stevenson DW (2006). ESTimating plant phylogeny: lessons from partitioning.. BMC Evol Biol.

[26] Bourlat SJ, Juliusdottir T, Lowe CJ, Freeman R, Aronowicz J, Kirschner M, Lander ES, Thorndyke M, Nakano H, Kohn AB, Heyland A, Moroz LL, Copley RR, Telford MJ (2006). Deuterostome phylogeny reveals monophyletic chordates and the new phylum Xenoturbellida.. Nature.

[27] Hughes J, Longhorn SJ, Papadopoulou A, Theodorides K, de Riva A, Mejia-Chang M, Foster PG, Vogler AP (2006). Dense taxonomic EST sampling and its applications for molecular systematics of the Coleoptera (beetles).. Mol Biol Evol.

[28] Benton MJ, Donoghue PCJ (2006). Paleontological Evidence to Date the Tree of Life.. MBE Advance Access published on October 17.

[29] Amrine-Madsen H, Scally M, Westerman M, Stanhope MJ, Krajewski C (2003). Nuclear gene sequences provide evidence for the monophyly of australidelphian marsupials.. Mol Phylogenet Evol..

[30] Jansa SA, Forsman JF, Voss RS (2006). Different patterns of selection on the nuclear genes IRBP and DMP-1 affect the efficiency but not the outcome of phylogeny estimation for didelphid marsupials.. Mol Phylogenet Evol.

[31] Watson JD, Hopkins NH, Roberts JW, Steitz AJ, Weiner AM (1965). Molecular biology of the gene..

[32] Warrington JA, Nair A, Mahadevappa M, Tsyganskaya M (2000). Comparison of human adult and fetal expression and identification of 535 housekeeping/maintenance genes.. Physiol Genomics.

[33] Hsiao LL, Dangond F, Yoshida T, Hong R, Jensen RV (2001). A compendium of gene expression in normal human tissues.. Physiol Genomics.

[34] Zheng XH, Lu F, Wang ZY, Zhong F, Hoover J (2005). Using shared genomic synteny and shared protein functions to enhance the identification of orthologous gene pairs.. Bioinformatics.

[35] Parkinson J, Guiliano DB, Blaxter M (2002). Making sense of EST sequences by CLOBBing them.. BMC Bioinformatics 2002.

[36] Endo T, Ikeo K, Gojobori T (1996). Large-Scale search for Genes on Which Positive selection May Operate.. Mol Biol Evol.

[37] Nei M, Gojobori T (1986). Simple methods for estimating the numbers of synonymous and nonsynonymous nucleotide substitutions.. Mol Biol Evol.

[38] Pie MR (2006). The Influence of Phylogenetic Uncertainty on the Detection of Positive Darwinian Selection.. Mol Biol Evol.

[39] The ENCODE Consortium (2007). The ENCODE pilot project: Functional annotation of 1% of the human genome.. Nature.

[40] Kjer KM, Honeycutt RL (2007). Site specific rates of mitochondrial genomes and the phylogeny of eutheria.. BMC Evol Biol.

[41] Cannarozzi GM, Schneider A, Gonnet G (2006). A Phylogenomic Study of Human, Dog and Mouse.. PLoS Computational Biology, e2.eor doi:10.1371/journal.pcbi.0030002.eor.

[42] Huttley GA, Wakefield MJ, Easteal S (2007). Rates of genome evolution and branching order from whole genome analysis.. Mol Biol Evol In press.

[43] Hughes AL, Friedman R (2007). The Effect of Branch Lengths on Phylogeny: an Empirical Study Using Highly Conserved Orthologs from Mammalian Genomes, Mol Phylogenet Evol doi:10.1016/j.ympev.2007.04.022.

[44] Felsenstein J (1978). Cases in which parsimony and compatibility methods will be positively misleading.. Syst Zool.

[45] Sullivan J, Swofford DL (1997). Are Guinea pigs rodents? The importance of adequate models in molecular phylogenetics.. J Mammal Evol.

[46] Baurain D, Brinkmann H, Philippe H (2006). Lack of resulution in the animal phylogeny: closely spaced cladogeneses or undetected systematic errors?. Mol Biol Evol In press.

[47] Yang Z (2006). Computional molecular evolution..

[48] Chomczynski P, Sacchi N (1987). Single-step method of RNA isolation by acid guanidinium thiocyanate-phenol-chloroform extraction.. Anal Biochem.

[49] NCBI RefSeq database ftp://ftp.ncbi.nih.gov/genomes.

[50] ENSEMBL public ftp ftp://ftp.ensembl.org/pub/.

[51] Thompson JD, Higgins DG, Gibson TJ (1994). CLUSTAL W: improving the sensitivity of progressive multiple sequence alignment through sequence weighting, positions-specific gap penalties and weight matrix choice.. Nucleic Acids Research,.

[52] Rambaut A (1996). Se-Alv2.0a11: sequence aignment editor.. http://evolve.zoo.ox.ac.uk/.

[53] Thomas PD, Campbell MJ, Kejariwal A, Mi H, Karlak B (2003). PANTHER: A Library of Protein Families and Subfamilies Indexed by Function.. Genome Res.

[54] Mi H, Lazareva-Ulitsky B, Loo R, Kejariwal A, Vandergriff J (2005). The PANTHER database of protein families, subfamilies, functions and pathways Nucl.. Acids Res.

[55] Strimmer K, von Haeseler A (1996). Quartet puzzling: a quartet maximum likelihood method for reconstructing tree topologies.. Mol Biol Evol.

[56] Felsenstein J (1993). PHYLIP (Phylogeny Inference Package) version 3.5c. Distributed by the author..

[57] Adachi J, Hasegawa M (1996). MOLPHY Version 2.3: Programs for molecular phylogenetics based on maximum likelihood.. Computer Science Monographs.

[58] Huelsenbeck JP, Ronquist F (2001). MRBAYES: Bayesian inference of phylogenetic trees.. Bioinformatics.

[59] Yang Z (1997). PAML: a program package for phylogenetic analysis by maximum lielihood.. Computer Applications in BioSciences.

[60] Jobb G, von Haeseler A, Strimmer K (2004). TREEFINDER: A powerful graphical analysis environment for molecular phylogenetics.. BMC Evol Biol.

[61] Guindon S, Gascuel S (2003). A simple, fast and accurate algorithm to estimate large phylogenies by maximum likelihood.. Syst Biol.

[62] Swofford DL (2002). PAUP* Phylogenetic Analysis using Parsimony(*and other Methods), version 4.0b8..

[63] Posada D, Crandall KA (1998). Modeltest: testing the model of DNA substitution.. Bioinformatics.

[64] Abascal F, Zardoya R, Posada D (2005). ProtTest: selection of the best-fit models of protein evolution.. Bioinformatics.

[65] Jones DT, Taylor WR, Thornton JM (1992). The rapid generation of mutation data matrices from protein sequences.. CABIOS.

[66] Lanave C, Preparata G, Saccone C, Serio G (1984). A new method for calculating evolutionary substitution rates.. J Mol Evol.

[67] Gu X, Fu YX, Li W-H (1995). Maximum likelihood estimation of the heterogeneity of substitution rate among nucleotide sites.. Mol Biol Evol.

[68] The Bioportal at University of Oslo.. http://www.bioportal.uio.no.

[69] Zhang J, Nielsen R, Yang Z (2005). Evaluation of an improved branch-site likelihood method for detecting positive selection at the molecular level.. Mol Biol Evol.

[70] Yang Z, Wong WS, Nielsen R (2005). Bayes empirical bayes inference of amino acid sites under positive selection.. Mol Biol Evol.

