# Supplementary material for: Expressed Sequence Tags as a Tool for Phylogenetic Analysis of Placental Mammal Evolution
Source: PLoS One. 2007 Aug 22;2(8):e775. doi: 10.1371/journal.pone.0000775 (PMC1942079; doi:10.1371/journal.pone.0000775)
Supplement: Table S1 — Accession numbers of 455 the human genes homologous to the ESTs (0.12 MB DOC) [file pone.0000775.s001.doc]

Supplementary Table S1. Accession numbers of 455 the human genes homologous to the ESTs

| NM_000075 | NM_000175 | NM_000284 | NM_000291 | NM_000391 | NM_000399 | NM_000428 |
| --- | --- | --- | --- | --- | --- | --- |
| NM_000546 | NM_000602 | NM_000637 | NM_000661 | NM_000687 | NM_000801 | NM_000821 |
| NM_000884 | NM_000903 | NM_000942 | NM_000967 | NM_000968 | NM_000969 | NM_000970 |
| NM_000975 | NM_000976 | NM_000978 | NM_000979 | NM_000980 | NM_000981 | NM_000983 |
| NM_000984 | NM_000985 | NM_000986 | NM_000988 | NM_000989 | NM_000990 | NM_000991 |
| NM_000992 | NM_000993 | NM_000994 | NM_000996 | NM_000997 | NM_000999 | NM_001000 |
| NM_001001342 | NM_001001479 | NM_001001563 | NM_001002913 | NM_001004333 | NM_001005 | NM_001006 |
| NM_001008741 | NM_001009 | NM_001011 | NM_001013 | NM_001014 | NM_001015 | NM_001017 |
| NM_001018 | NM_001020 | NM_001021 | NM_001022 | NM_001024 | NM_001025 | NM_001028 |
| NM_001030 | NM_001031 | NM_001032 | NM_001064 | NM_001098 | NM_001126 | NM_001129 |
| NM_001183 | NM_001212 | NM_001269 | NM_001284 | NM_001311 | NM_001312 | NM_001404 |
| NM_001414 | NM_001425 | NM_001428 | NM_001436 | NM_001451 | NM_001456 | NM_001487 |
| NM_001605 | NM_001614 | NM_001625 | NM_001628 | NM_001636 | NM_001685 | NM_001689 |
| NM_001697 | NM_001743 | NM_001749 | NM_001752 | NM_001895 | NM_001902 | NM_001904 |
| NM_001916 | NM_001948 | NM_001959 | NM_001961 | NM_001970 | NM_001997 | NM_002046 |
| NM_002087 | NM_002096 | NM_002105 | NM_002106 | NM_002131 | NM_002137 | NM_002273 |
| NM_002295 | NM_002306 | NM_002394 | NM_002415 | NM_002467 | NM_002496 | NM_002574 |
| NM_002624 | NM_002634 | NM_002708 | NM_002743 | NM_002746 | NM_002778 | NM_002791 |
| NM_002796 | NM_002798 | NM_002804 | NM_002808 | NM_002813 | NM_002824 | NM_002910 |
| NM_002915 | NM_002923 | NM_002948 | NM_002949 | NM_002954 | NM_003073 | NM_003127 |
| NM_003132 | NM_003271 | NM_003295 | NM_003299 | NM_003329 | NM_003347 | NM_003387 |
| NM_003418 | NM_003461 | NM_003491 | NM_003564 | NM_003567 | NM_003670 | NM_003746 |
| NM_003754 | NM_003761 | NM_003779 | NM_003827 | NM_003846 | NM_003849 | NM_003860 |
| NM_003883 | NM_003900 | NM_003905 | NM_003945 | NM_004068 | NM_004135 | NM_004147 |
| NM_004175 | NM_004309 | NM_004326 | NM_004328 | NM_004343 | NM_004450 | NM_004483 |
| NM_004515 | NM_004559 | NM_004596 | NM_004710 | NM_004763 | NM_004766 | NM_004846 |
| NM_004879 | NM_004901 | NM_004907 | NM_004924 | NM_005004 | NM_005005 | NM_005008 |
| NM_005022 | NM_005030 | NM_005053 | NM_005087 | NM_005094 | NM_005105 | NM_005111 |
| NM_005166 | NM_005176 | NM_005230 | NM_005262 | NM_005324 | NM_005438 | NM_005500 |
| NM_005507 | NM_005530 | NM_005548 | NM_005570 | NM_005600 | NM_005617 | NM_005620 |
| NM_005625 | NM_005662 | NM_005669 | NM_005718 | NM_005719 | NM_005781 | NM_005801 |
| NM_005830 | NM_005866 | NM_005877 | NM_005917 | NM_005998 | NM_006013 | NM_006098 |
| NM_006122 | NM_006145 | NM_006191 | NM_006234 | NM_006253 | NM_006280 | NM_006305 |
| NM_006312 | NM_006324 | NM_006325 | NM_006347 | NM_006409 | NM_006429 | NM_006442 |
| NM_006449 | NM_006487 | NM_006503 | NM_006519 | NM_006530 | NM_006571 | NM_006597 |
| NM_006601 | NM_006628 | NM_006666 | NM_006667 | NM_006698 | NM_006708 | NM_006739 |
| NM_006755 | NM_006761 | NM_006830 | NM_006836 | NM_006857 | NM_006899 | NM_006937 |
| NM_007006 | NM_007104 | NM_007234 | NM_007263 | NM_007270 | NM_007273 | NM_007355 |
| NM_007363 | NM_012111 | NM_012181 | NM_012225 | NM_012232 | NM_012311 | NM_012382 |
| NM_012425 | NM_012458 | NM_013234 | NM_013265 | NM_013328 | NM_014017 | NM_014041 |
| NM_014062 | NM_014078 | NM_014187 | NM_014300 | NM_014302 | NM_014325 | NM_014339 |
| NM_014390 | NM_014409 | NM_014453 | NM_014463 | NM_014502 | NM_014574 | NM_014604 |
| NM_014718 | NM_014764 | NM_014787 | NM_014814 | NM_014972 | NM_015150 | NM_015157 |
| NM_015187 | NM_015231 | NM_015292 | NM_015380 | NM_015414 | NM_015440 | NM_015496 |
| NM_015629 | NM_015680 | NM_015703 | NM_015710 | NM_015913 | NM_015920 | NM_016047 |
| NM_016078 | NM_016118 | NM_016139 | NM_016145 | NM_016155 | NM_016185 | NM_016223 |
| NM_016306 | NM_016319 | NM_016390 | NM_016400 | NM_016491 | NM_016609 | NM_017570 |
| NM_017739 | NM_018174 | NM_018178 | NM_018221 | NM_018285 | NM_018361 | NM_018394 |
| NM_018622 | NM_019008 | NM_019037 | NM_019070 | NM_019071 | NM_019073 | NM_019087 |
| NM_020122 | NM_020196 | NM_020216 | NM_020441 | NM_021009 | NM_021029 | NM_021968 |
| NM_021975 | NM_022719 | NM_022821 | NM_022916 | NM_024005 | NM_024071 | NM_024078 |
| NM_024085 | NM_024092 | NM_024295 | NM_024300 | NM_024321 | NM_024333 | NM_024506 |
| NM_024520 | NM_024536 | NM_024656 | NM_025029 | NM_025164 | NM_025238 | NM_030900 |
| NM_031157 | NM_031266 | NM_031314 | NM_031844 | NM_031966 | NM_032014 | NM_032111 |
| NM_032309 | NM_032338 | NM_032353 | NM_032378 | NM_032506 | NM_032525 | NM_032826 |
| NM_033301 | NM_033362 | NM_033419 | NM_033452 | NM_033453 | NM_033557 | NM_052837 |
| NM_052848 | NM_052875 | NM_053024 | NM_053275 | NM_079423 | NM_080667 | NM_106552 |
| NM_130445 | NM_130787 | NM_138391 | NM_138412 | NM_138578 | NM_139034 | NM_144582 |
| NM_144772 | NM_145040 | NM_145048 | NM_145266 | NM_145269 | NM_145729 | NM_152265 |
| NM_152391 | NM_152415 | NM_152440 | NM_152524 | NM_152653 | NM_153218 | NM_170707 |
| NM_173084 | NM_173642 | NM_173794 | NM_174913 | NM_175058 | NM_176794 | NM_178564 |
| NM_178581 | NM_178863 | NM_181715 | NM_182569 | NM_184043 | NM_194460 | NM_198086 |
| NM_198216 | NM_198244 | NM_198318 | NM_198437 | NM_198889 | NM_199040 | NM_199185 |
| NM_199417 | NM_201554 | NM_203364 | NM_206538 | NM_207013 | NM_212482 | XM_085634 |
| XM_114317 | XM_208072 | XM_370992 | XM_495885 | XM_496225 | XM_496555 | XM_496612 |
